# Widespread Misinterpretable ChIP-seq Bias in Yeast

**DOI:** 10.1371/journal.pone.0083506

**Published:** 2013-12-09

**Authors:** Daechan Park, Yaelim Lee, Gurvani Bhupindersingh, Vishwanath R. Iyer

**Affiliations:** Center for Systems and Synthetic Biology, Institute for Cellular and Molecular Biology, Department of Molecular Biosciences, University of Texas, Austin, Texas, United States of America; CRG, Spain

## Abstract

Chromatin immunoprecipitation followed by sequencing (ChIP-seq) is widely used to detect genome-wide interactions between a protein of interest and DNA *in vivo*. Loci showing strong enrichment over adjacent background regions are typically considered to be sites of binding. Insufficient attention has been given to systematic artifacts inherent to the ChIP-seq procedure that might generate a misleading picture of protein binding to certain loci. We show here that unrelated transcription factors appear to consistently bind to the gene bodies of highly transcribed genes in yeast. Strikingly, several types of negative control experiments, including a protein that is not expected to bind chromatin, also showed similar patterns of strong binding within gene bodies. These false positive signals were evident across sequencing platforms and immunoprecipitation protocols, as well as in previously published datasets from other labs. We show that these false positive signals derive from high rates of transcription, and are inherent to the ChIP procedure, although they are exacerbated by sequencing library construction procedures. This expression bias is strong enough that a known transcriptional repressor like Tup1 can erroneously appear to be an activator. Another type of background bias stems from the inherent nucleosomal structure of chromatin, and can potentially make it seem like certain factors bind nucleosomes even when they don't. Our analysis suggests that a mock ChIP sample offers a better normalization control for the expression bias, whereas the ChIP input is more appropriate for the nucleosomal periodicity bias. While these controls alleviate the effect of the biases to some extent, they are unable to eliminate it completely. Caution is therefore warranted regarding the interpretation of data that seemingly show the association of various transcription and chromatin factors with highly transcribed genes in yeast.

## Introduction

The genome-wide mapping of protein localization on chromatin at high resolution is crucial for understanding the molecular mechanisms of transcription *in vivo*. Chromatin immunoprecipitation (ChIP) followed by deep sequencing (ChIP-seq) is currently the preferred and widespread method to accomplish this [1–3]. Because of the power of the ChIP assay, the Encyclopedia of DNA Elements (ENCODE) and Roadmap Epigenome Projects have adopted ChIP-seq to map the genomic locations of many transcription factors, histone marks, and DNA modifications in both cell lines and model organisms [4–7]. Because the localization of chromatin-associated factors is dependent on cell type and environmental conditions [8,9], ChIP-seq is being increasingly used to explore hundreds of DNA-binding proteins in different types of cells and under different conditions.

Yeast is the first and only eukaryote for which nearly every transcription factor has been ChIP-ed and for which the resulting immunoprecipitated DNA has been mapped on a genome-wide scale using microarrays [10,11]. With the advent of deep sequencing technology, ChIP-seq also has been broadly applied to yeast genomics [12–14]. Yeast is ideal for comprehensive studies on protein-DNA interactions due to its relatively small genome, the resulting low cost of experiments, and the availability of a tandem affinity purification (TAP)-tagged collection for 80% of its proteins [15]. This latter benefit is of particular importance, as TAP-tagged strains do not suffer from the same non-uniform quality as antibodies, whose variability can affect the efficiency of ChIP.

Several algorithms have been developed to computationally identify peaks of enrichment in ChIP-seq data, indicative of protein binding locations, and to distinguish such peaks from background reads [1,16]. Experimentally and computationally, the background signal is typically defined using either a parallel input sample which has not been subject to the immunoprecipitation step, after reversal of crosslinks, or a mock ChIP sample (where a non-specific IgG antibody, or pre-immune serum, or an untagged strain is used).

In the course of carrying out ChIP-seq experiments for various yeast transcription-related proteins, we unexpectedly found strong enrichment signals suggestive of proteins binding to genomic loci where genes were highly transcribed, regardless of which protein was being analyzed. The functions of the genes exhibiting this universally high protein occupancy however did not always align with the established roles of the proteins apparently binding to them. Moreover, the enrichment for proteins binding to highly-transcribed genes was observed even in controls like mock ChIP-seq data, which points to an overall bias that could contaminate any ChIP-seq data with false positives. A secondary bias of nucleosomal periodicity was also commonly observed across ChIP-seq datasets and contributed additional false positives in which proteins falsely appeared to interact with nucleosomes. We present our analysis of this phenomenon, and suggest ways in which these artifacts can be ameliorated by the proper choice of control experiments. Our data suggest however that the enrichment bias at highly transcribed genes could be an intrinsic characteristic of ChIP-seq experiments, and caution is therefore warranted in interpreting the results of ongoing and published results purporting to show the association of many proteins with the transcribed regions of genes.

## Materials and Methods

### Yeast strains and culture conditions

The yeast strain used in this study as a WT was BY4741 (*MATa his3Δ1 leu2Δ0 met15Δ0 ura3Δ0*). For ChIP, the TAP-tagged yeast strains including SWI6-TAP, TUP1-TAP, RSC2-TAP and MNN10-TAP strains were obtained from the yeast TAP-fusion collection (Open Biosystems) [15]. We generated the HSF1-TAP strain from BY4741 by integrating the TAP-HIS3MX6 cassette into the 3'-end of *HSF1* through homologous recombination, enabling the expression of C-terminal TAP-tagged Hsf1. Using the same scheme, we also generated a SWI6-13XMYC strain from BY4741. For gene expression profiling, we used the *TUP1* deletion strain from the yeast deletion collection (Open Biosystems) [17]. The identity of all engineered strains was verified by genomic PCR. Normal growth conditions were 30°C in YPD (Yeast extract, peptone, dextrose) media with shaking at 250 rpm. Yeast cells were grown to mid-log phase (O.D 600 nm of 0.6 to 0.8), fixed with formaldehyde and collected for ChIP; or, were collected without fixation for gene expression profiling. For heat shock, mid-log phase yeast cells were collected and re-suspended in pre-warmed 39°C YPD media, then incubated for 15 min at 39°C. For rapamycin treatment, either DMSO or rapamycin was added to mid-log phase yeast cells and incubated for 30 min at 30°C. Since DMSO is a solvent for rapamycin, control and rapamycin-added cells were treated with DMSO and rapamycin to be a final concentration of 0.1% and 100 nM, respectively.

### Chromatin immunoprecipitation

Proteins were crosslinked to DNA by adding formaldehyde to the culture (final concentration of 1%). Crosslinking was done for 15 min and quenched with glycine (final concentration of 0.125 M) for 5 min. Yeast cells were re-suspended with lysis buffer and disrupted by agitation with glass beads using a Bead beater (BioSpec Products). The cell lysates were sheared using a Branson Sonifier (Emerson Industrial Automation), and immunoprecipitated using the following beads or antibody: IgG Sepharose 6 Fast Flow (GE Healthcare Life Sciences) to pull-down all TAP-tagged proteins used in this study, anti-Myc conjugated agarose bead (Sigma Aldrich, cat.# E6654) to pull-down Swi6 in the SWI6-13XMyc strains, and RNAPII Ser5P antibody (Abcam, cat.# ab5131) to pull-down active RNAPII. Mock ChIP DNA was prepared by immunoprecipitation with IgG Sepharose in the wild type strain with no TAP-tagged protein expression. Input DNA was prepared in parallel with the SWI6-TAP ChIP sample but leaving out the immunoprecipitation step. The crosslinks were reversed by heating at 65°C for 12-16 hours and the immunoprecipitated DNA was purified using UltraPure Phenol:Chloroform:Isoamyl alcohol (25:24:1 v/v, Invitrogen).

### Sequencing library preparation

Sequencing library preparation with ChIP-ed DNA and input DNA was carried out by following either the NEB ChIP-seq library preparation for Illumina (New England Biolabs) or the SOLiD V3 barcoded fragment library preparation protocol (Life Technologies). Sequencing was performed through either Illumina HiSeq 2000 or SOLiD V4 at the University of Texas at Austin Genome Sequencing and Analysis Facility.

### Gene expression profiling

The collected yeast cells were re-suspended with AE buffer (50 mM Sodium Acetate pH 5.2, 10 mM EDTA) containing 1.7% SDS, and total RNA was extracted with a hot acid phenol method [18]. Double-stranded cDNA was synthesized from total RNA, and labeled with Cy3 using the NimbleGen One-Color DNA labeling kits (Roche NimbleGen). The labeled cDNA was hybridized onto a NimbleGen *S. cerevisiae* HX12 array (Roche NimbleGen), and the array was washed and scanned with a GenePix 4000B scanner (Molecular Devices). The scanned image was processed using NimbleScan for quantification of signal intensities and Robust Multi-array Average normalization with a large set of other NimbleGen array datasets in our lab (Roche NimbleGen). Differentially expressed genes in *tup1∆* relative to WT were identified with Bioconductor limma package version 3.14.4**.**

### Quantitative PCR

Three high TR genes (*CCW12*, *TDH3*, and *PDC1*) and three low TR genes (*PDR8*, *HKR1*, and *BIT61*) were selected. Two control primers used for normalization were designed from the tail-to-tail intergenic regions between YHL004W (*MRP4*) and YHL003C, stated as iYHL004W, and between YCR023C and YCR024C, described as iYCR024C. Primer pairs used in qPCR were designed to amplify 80-100 bp regions within the respective ORFs, and their sequences are provided in Table S1. qPCR was performed using Power SYBR Green PCR Master Mix (Applied Biosystems) on a ViiA7 Real Time PCR System (Life Technologies). For relative quantification of target DNA compared to control DNA, qPCR data was analyzed through a standard curve-based method.

### Deep sequencing data analysis

Deep sequencing data were mapped onto the unmasked sacCer3 reference (http://hgdownload.cse.ucsc.edu/goldenPath/sacCer3/bigZips/chromFa.tar.gz ) using BWA (Version: 0.5.9-r16) with default options [19]. Non-uniquely mapped reads were filtered out in order to remove reads with low mapping quality. Wig files of sequencing data were loaded in a local mirror of the UCSC Genome Browser for snapshots [20]. For average read profiles, reads were counted by bin size 10 bp within 1.5 kb from transcription start sites (unpublished data), and counts were divided by the total number of mapped reads and multiplied by 1 million. The graphs were plotted either using the standard Python library and package matplotlib and numpy [21], R or Microsoft Excel. Scripts are available upon request. Peak calling was performed with MACS2 (version: 2.0.9) [22]. Cse4 and untagged control ChIP-seq were downloaded from Gene Expression Omnibus database (GEO) Series accession number GSE13322 and GSE20870 [13,23], respectively. We also downloaded histone MNase ChIP-seq data from NCBI Sequence Read Archive accession number SRA012303 [24]. These published datasets were processed with the same analysis pipeline as above.

### Mock and input comparison

We executed the MACS2 module (version: 2.0.9) for 4 different experimental pairs: 1) DMSO Tup1 ChIP and DMSO input, 2) DMSO Tup1 ChIP and DMSO mock ChIP, 3) Rap Tup1 ChIP and Rap input, and 4) Rap Tup1 ChIP and Rap mock ChIP. Also, two thresholds (-log_10_(q-value) = 2 and 20) were chosen to compare the efficiency of a threshold to eliminate expression bias peaks based upon stringency (Table 1). Then, MAnorm was utilized to identify differential binding targets (DBTs) from the MACS generated data [25]. These programs were run with default parameters, with the fragment extension length being set to 75 bases. MAnorm allowed us to ignore regions where the control showed higher signals than the treated sample. Thus, by using different controls in MACS followed by MAnorm analysis, we were able to test the effect of controls on the removal of background signals based on the number of DBTs and the percentage of DBTs within gene bodies. We transferred the MACS peak data from experimental pairs 1 and 3 (see above) to MAnorm and repeated for experimental pairs 2 and 4 (see above). We applied the same cut-off p-value (-log_10_(q-value) = 5) for DBTs to the MAnorm results.

**Table 1:** Rapamycin-specific Tup1 peaks using MACS followed by MAnorm analysis.

**Category**		**Cut-off Stringency**	**Input Correction**	**Mock Correction**
MACS2 peak calling	Control	Low	2478	726
		High	1120	296
	Rapamycin	Low	2419	845
		High	725	309
Rapamycin-specific targets by MAnorm		Low	770	407
		High	384	165
Rapamycin-specific targets within gene bodies		Low	379 (49.2%)	149 (36.6%)
		High	139 (36.2%)	32 (19.4%)

Low and high stringency cut-offs were -log_10_(q-value) = 2 and 20, respectively. Rapamycin-specific targets were those differential binding peaks found by MAnorm with -log_10_(q-value) > 5.

### Data availability

Sequencing data reported in this manuscript are available from NCBI GEO as GSE51251 (http://www.ncbi.nlm.nih.gov/geo/query/acc.cgi?acc=GSE51251) and microarray expression profiling data are available under GSE51376 (http://www.ncbi.nlm.nih.gov/geo/query/acc.cgi?acc=GSE51376).

## Results

### Common enrichment signals appear in ChIP-seq datasets spanning factors, growth conditions, and sequencing platforms

In order to study the targets of chromatin binding proteins in response to transcriptional perturbations, we performed ChIP-seq against multiple chromatin-associated factors after treatment of cells with rapamycin (with DMSO treatment serving as control) or heat shock (at 39°C, with growth at 30°C serving as control). Included among these experiments were two unrelated transcription factors, Swi6 and Tup1, and various negative controls. One type of control was a mock ChIP-seq, in which immunoglobulin G (IgG)-conjugated sepharose beads were incubated with wild-type (WT) yeast chromatin. In another control, the input of a Swi6 ChIP sample (the sheared chromatin from a SWI6 TAP-tagged strain) was sequenced. Finally, we also ChIP-ed a subunit of Golgi mannosyltransferase complex Mnn10; as a cytoplasmic complex, Mnn10 is unlikely to associate with chromatin and thus was not expected to pull down any DNA.

**Figure 1:**
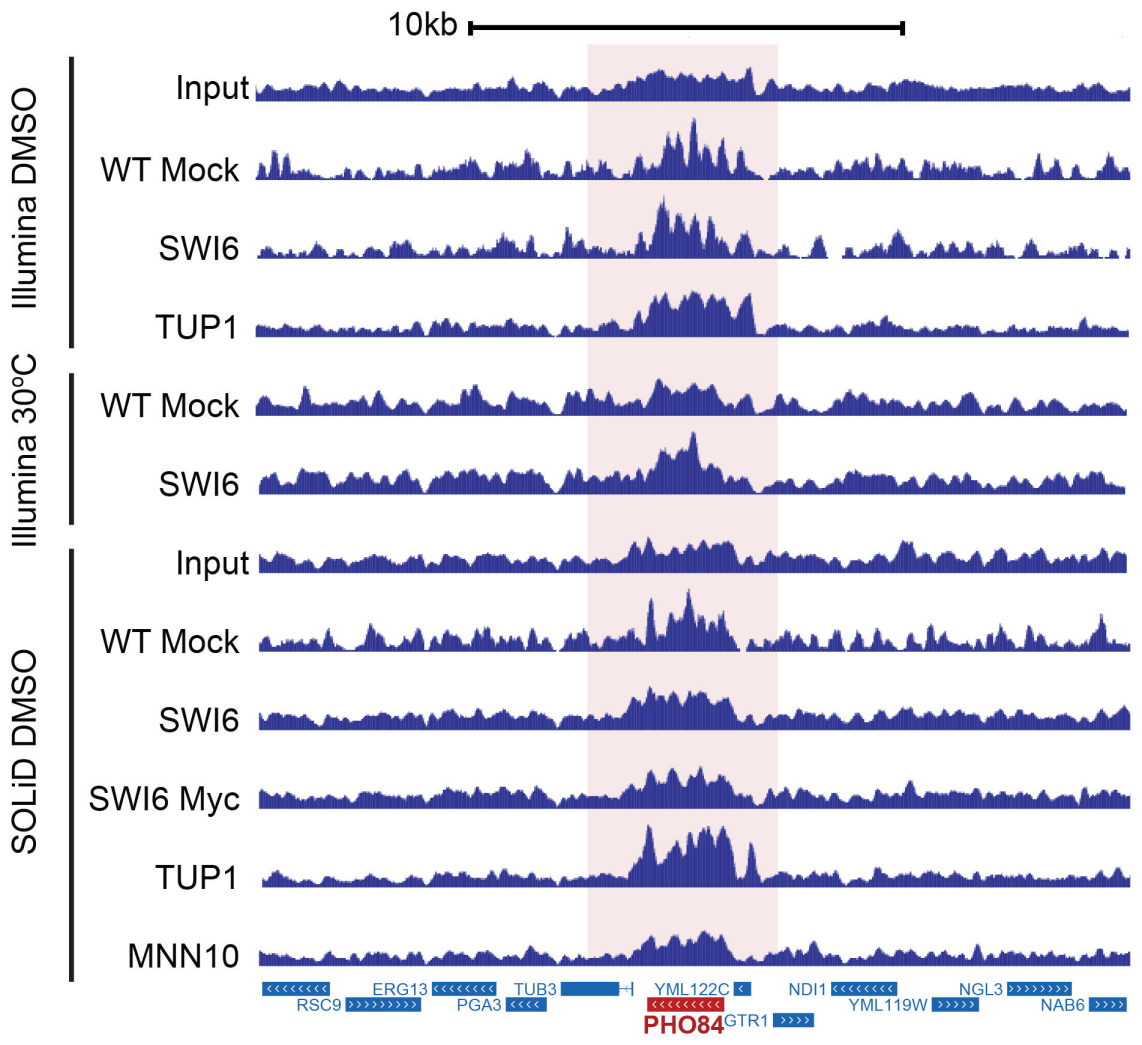
Example of high background signal across multiple datasets. Sequencing datasets from different factors, controls, epitope tags, transcription factors and growth conditions as indicated are represented in a browser view. Based on the read counts normalized by transcript lengths from RNA-seq data [44], PHO84 is the 82^nd^ most highly expressed gene under normal conditions in WT yeast.

We noticed that surprisingly, common targets were enriched across several data sets, including Mnn10 ChIP (Figure 1). Such peaks were observed across different sequencing platforms (Illumina or SOLiD), epitope tags (SWI6 TAP-tagged or SWI6 13XMyc tagged), bead types (IgG-tagged sepharose beads or c-Myc antibody-conjugated agarose beads), and immunoprecipitated factors (Swi6 or Tup1) (Figure S1), indicating that the shared signals were not derived from the use of a specific protocol or reagent. Perhaps most significantly, the targets were shared between the standard mock ChIP and input control experiments, suggesting that these shared targets represented non-random false positives.

### Highly expressed genes demonstrate widespread, strong ChIP-seq signals

We next examined whether the phenomenon described above was generally observable genome-wide. We observed two features among the strong false positive signals. First, the signals were present within gene bodies and second, the strongest signals derived from yeast genes that are known to be highly expressed. Thus, we termed this artifact an "expression bias". In order to better define the set of highly transcribed genes, we performed ChIP-seq against active RNA polymerase II under the same conditions. The occupancy of RNAPII phosphorylated at serine 5 of its C-terminal domain repeats (RNAPII Ser5P) is a better indicator of transcription rate than steady state RNA levels [26]. We defined the top 100 open reading frames (ORFs) in terms of RNAPII Ser5P occupancy (after normalizing for gene length and sequencing depth) as high transcription rate (high TR) genes.

**Figure 2:**
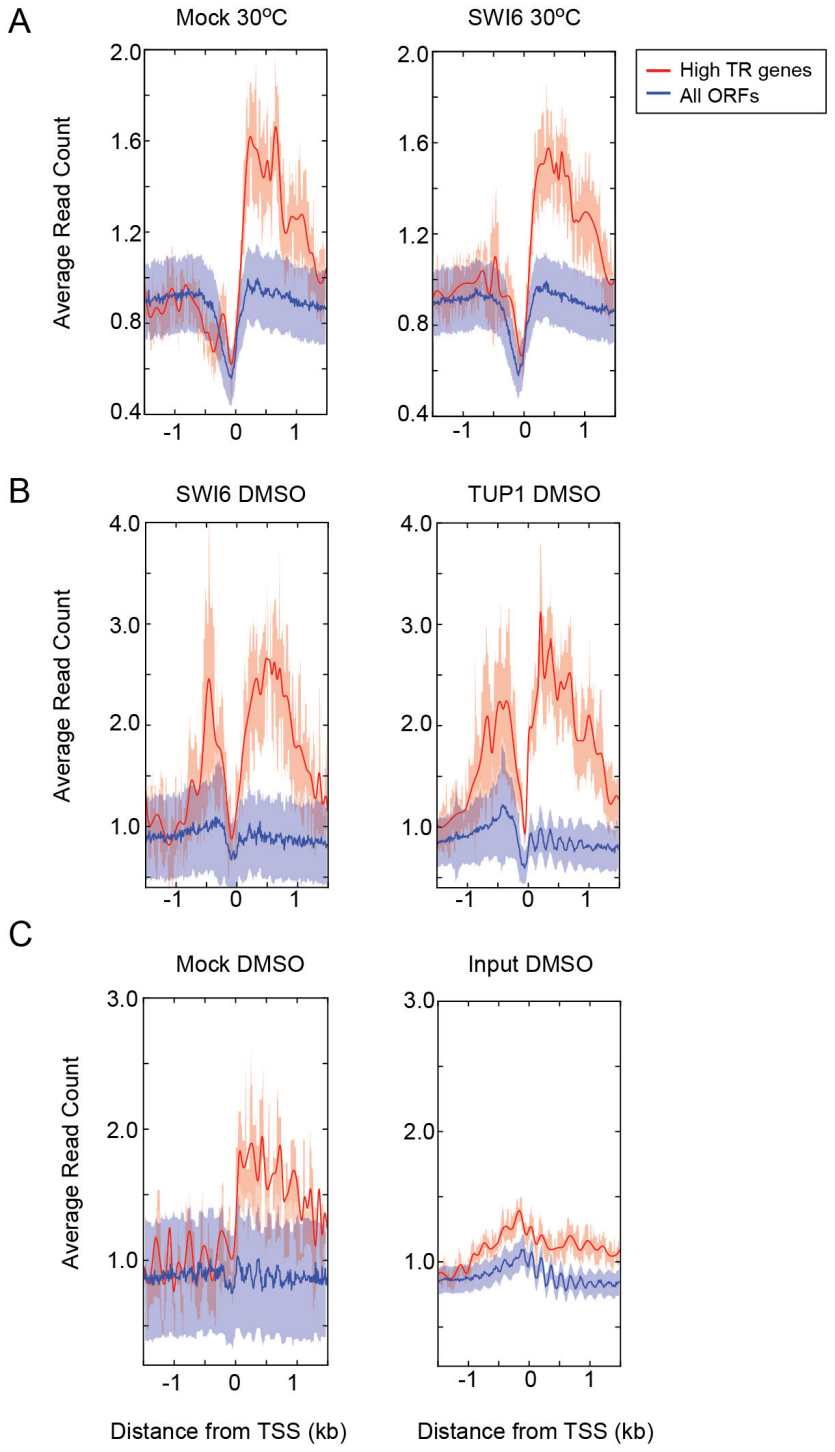
Genes with high transcription rates (TR) have high average read counts within gene bodies in ChIP-seq and control experiments. Lines show average read counts in 10 bp bins for the indicated groups of genes, which are either the 100 most highly transcribed genes based on RNAPII Ser5P occupancy as described in the text (High TR genes, red line) or all the other genes (All ORFs, blue line). The shaded bands represent the 95% confidence interval of the data. All ChIP samples in this figure were sequenced using the Illumina platform. (A) Under normal growth conditions (30°C in YPD), mock ChIP had comparable bias to Swi6 ChIP. (B) Both SWI6 (an activator) and TUP1 (a repressor) show comparable high levels of the expression bias at high TR genes. (C) Input has a lower expression bias than mock ChIP. For (B) and (C) cells were treated with DMSO, which was a control for rapamycin treatment.

Read counts over genes in several ChIP-seq and control experiments were strongly enriched for high TR genes compared to other genes (Figure 2A, B, and C). Consistent with the example shown in Figure 1, the expression bias was a recurrent artifact in all ChIP-seq data, although the degree of expression bias varied from factor to factor. To examine if the expression bias was an artifact specific to ChIP-seq data from our lab, we downloaded previously published ChIP-seq data from other labs and analyzed them using the same pipeline [13,23]. Specifically, we compared ChIP for a centromere binding protein, Cse4 [13], and an independent mock ChIP that had been used as a negative control for the association of the transcription factor Tbf1 [23]. Cse4 in particular is a centromere-specific histone H3 variant that is not expected to occupy transcribed regions. Both of these published datasets exhibited the same artifacts as we describe above (Figure S2), suggesting that the expression bias seen for high TR genes is a commonly occurring phenomenon in yeast ChIP-seq data and could confound the interpretation of many types of experiments.

### Expression bias of ChIP-seq data is exacerbated by condition-specific transcriptional activation

The transcript levels of stress-responsive genes are dramatically altered by rapamycin treatment and heat shock [27,28]. Given the fact that upregulated genes under stress conditions show comparable transcription rates to high TR genes under normal conditions, we wondered whether the expression bias in ChIP would similarly be detectable in upregulated genes specifically under stress conditions. To answer this question, we first measured the condition-specific occupancy of active RNAPII on chromatin by ChIP-seq after rapamycin treatment and heat shock. The top 100 ORFs showing increased occupancy after treatment relative to normal were defined as transcriptionally upregulated genes in response to rapamycin and heat shock (or “Rap Up” genes and “Heat Up” genes), respectively.

**Figure 3:**
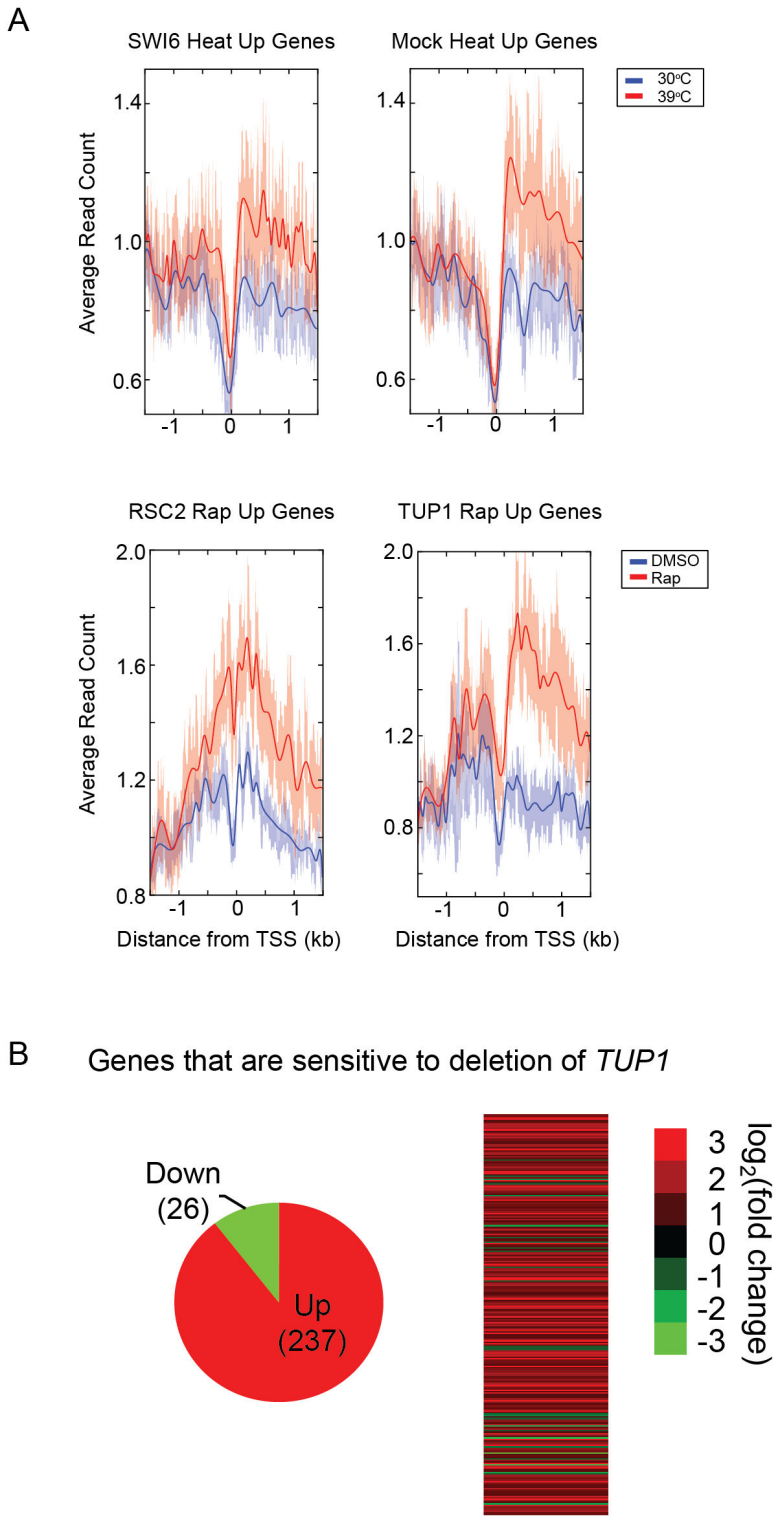
Condition-specific increase in apparent binding at genes that are transcriptionally activated. (A) ChIP-seq data for an activator (Swi6), co-repressor (Tup1), chromatin remodeler (Rsc2), and a mock ChIP control for genes that are transcriptionally activated by the indicated treatment. "Heat Up" are genes activated by heat shock, and "Rap Up" are genes activated by rapamycin treatment. Red lines show data after treatment (39°C or rapamycin), while blue lines show data before treatment (30°C or DMSO) for the same set of genes. (B) Differentially expressed genes comparing a WT strain to *tup1∆*. The majority of genes were activated upon deletion of TUP1, demonstrating that Tup1 is primarily a transcriptional repressor.

As the cell cycle is arrested at G1 by heat shock [29], we reasoned that Swi6, a well-known transcriptional activator of the G1/S transition [30,31], would not bind strongly to heat shock-induced genes. Surprisingly, we found that Swi6 bound strongly to the transcribed regions of Heat Up genes specifically after heat shock (Figure 3A). A mock ChIP control sample for this experiment showed similar enrichment at Heat Up genes. While this illustrated the expression bias as manifested for differentially expressed genes during a perturbation, we investigated a different stress condition to rule out the possibility that the expression bias was specific to heat shock or to Swi6. We performed ChIP-seq for Rsc2, a component of the RSC chromatin remodeling complex, and Tup1, a component of the TUP1-CYC8 co-repressor complex, after rapamycin treatment of cells. Both Rsc2 and Tup1 showed high occupancy over the transcribed regions of Rap Up genes after rapamycin treatment (Figure 3A). Thus, unrelated transcription factors appear to show increased binding to the ORFs of genes that are more actively transcribed after different environmental perturbations.

### Expression bias can give misleading information regarding the biological function of transcription factors

Despite the expression bias observed in mock ChIP and other control experiments above, it is possible that certain transcription factors also truly bind to ORFs as a means of regulating gene expression. For example, occupancy by a transcription factor of the ORFs of high TR genes, or of Heat Up genes specifically after heat shock might suggest a role in activating transcriptional elongation, something that cannot be formally ruled out based on our data for Swi6. However, the case of Tup1 offers a means of testing this notion. The molecular mechanism of the Tup1-Cyc8 complex as a general transcriptional repressor has been well established [32]. In order to confirm that Tup1 does not also serve as a transcriptional activator, we performed gene expression profiling of a *tup1Δ* strain compared to WT. Almost 90% of the differentially expressed genes were repressed by Tup1, showing that Tup1 does not, in fact, activate these genes in wild type cells (Figure 3B). Yet, ChIP-seq data for Tup1 suggested just the opposite. Tup1 occupied high TR genes as opposed to the low TR genes one would expect for a repressor. In this instance therefore, occupancy of high TR genes by Tup1 is likely to give a misleading picture regarding its biological function.

**Figure 4:**
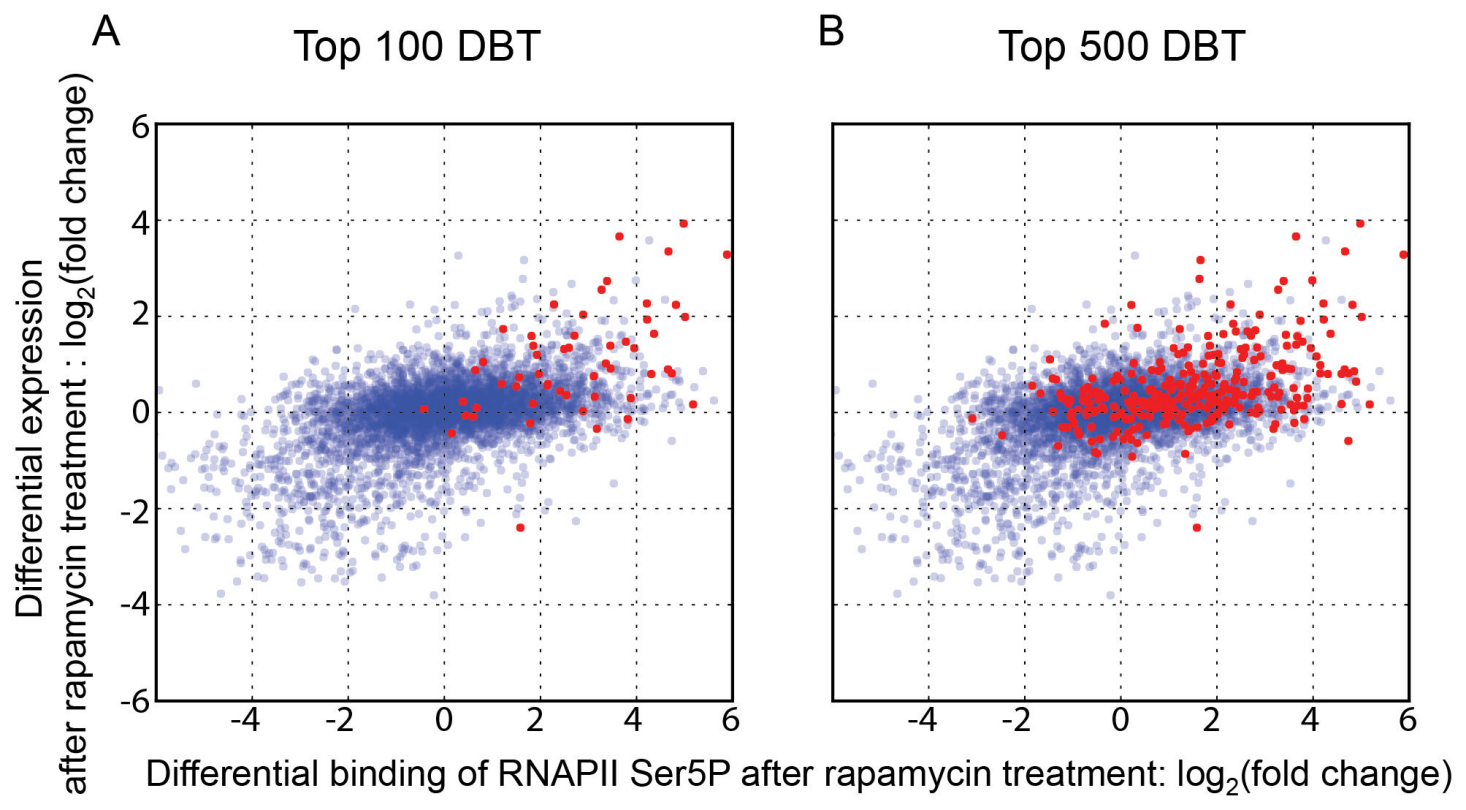
Uncorrected Tup1 differential binding targets misleadingly indicate that Tup1 is primarily a transcriptional activator. Scatter plots show the differential transcriptional activation after rapamycin treatment as blue points. Differential RNAPII Ser5P occupancy before and after rapamycin treatment was measured by ChIP-seq and plotted on the X-axis. Differential mRNA expression levels in the same cultures were measured using microarrays and plotted on the Y-axis, in scatter plots showing 4929 genes. We used MACS to identify differential binding targets (DBTs) of Tup1 as described in the text and plotted them on the same plots in red. (A) The top 100 DBT peaks ranked by fold change were assigned to 55 ORFs, which are plotted in red. (B) The top 500 DBT peaks were assigned to 295 ORFs, which are plotted in red. Tup1 DBT ORFs tended to be upregulated genes in response to rapamycin.

A common use of ChIP-seq is to examine binding of a given factor under different growth conditions or backgrounds. Since only a single variable is changed (the experimental or growth condition), it might be assumed that comparing binding under different conditions offers a reliable means of identifying biologically relevant targets, with most background artifacts being normalized out. We wondered whether the expression bias we noted earlier could nevertheless confound the interpretation of such experiments. We used the MACS algorithm to identify targets showing increased binding of Tup1 in response to rapamycin treatment [22]. We used vehicle (DMSO) treated cells as the control and rapamycin treated cells as the experimental sample, and used MACS to identify differential binding targets (DBTs) from the ChIP-seq data for Tup1 under these two parallel conditions. 57 of the top 100 and 322 of the top 500 DBTs identified by MACS were in ORFs. Strikingly, the majority of these DBT ORFs were ORFs that were transcriptionally activated by rapamycin treatment. When superimposed on a scatterplot of gene expression versus RNAPII Ser5P occupancy, the Tup1 DBT ORFs were concentrated in the upper right quadrant (Figure 4A and 4B). In the absence of other knowledge about Tup1 function, one would misinterpret this data to mean that Tup1, since it associates with the ORFs of rapamycin-upregulated genes after rapamycin treatment, likely functions in the activation of those genes. These results therefore raised the question of what type of normalization controls might be appropriate for minimizing false positives in ChIP-seq data, even when analyzing differential binding under different conditions.

### Mock ChIP is a better control for expression bias than ChIP input, but is not infallible

We observed that mock ChIP-seq data exhibited a stronger expression bias than the corresponding input samples (Figure 2C), and therefore hypothesized that correction by mock ChIP (normalization) would more effectively reduce the false-positives exemplified by Tup1 DBT ORFs than normalization by input. To test this hypothesis, we first used MACS to normalize each condition specific ChIP-seq dataset to either its corresponding input or mock ChIP-seq sample. We used low and high stringency thresholds to compare their effectiveness in minimizing false positives (Table 1). We then used MAnorm to identify DBTs from this MACS-normalized data [25]. At a given p-value threshold, fewer Tup1 DBTs were identified when using mock ChIP-seq data as the normalization control (Table 1).

**Figure 5:**
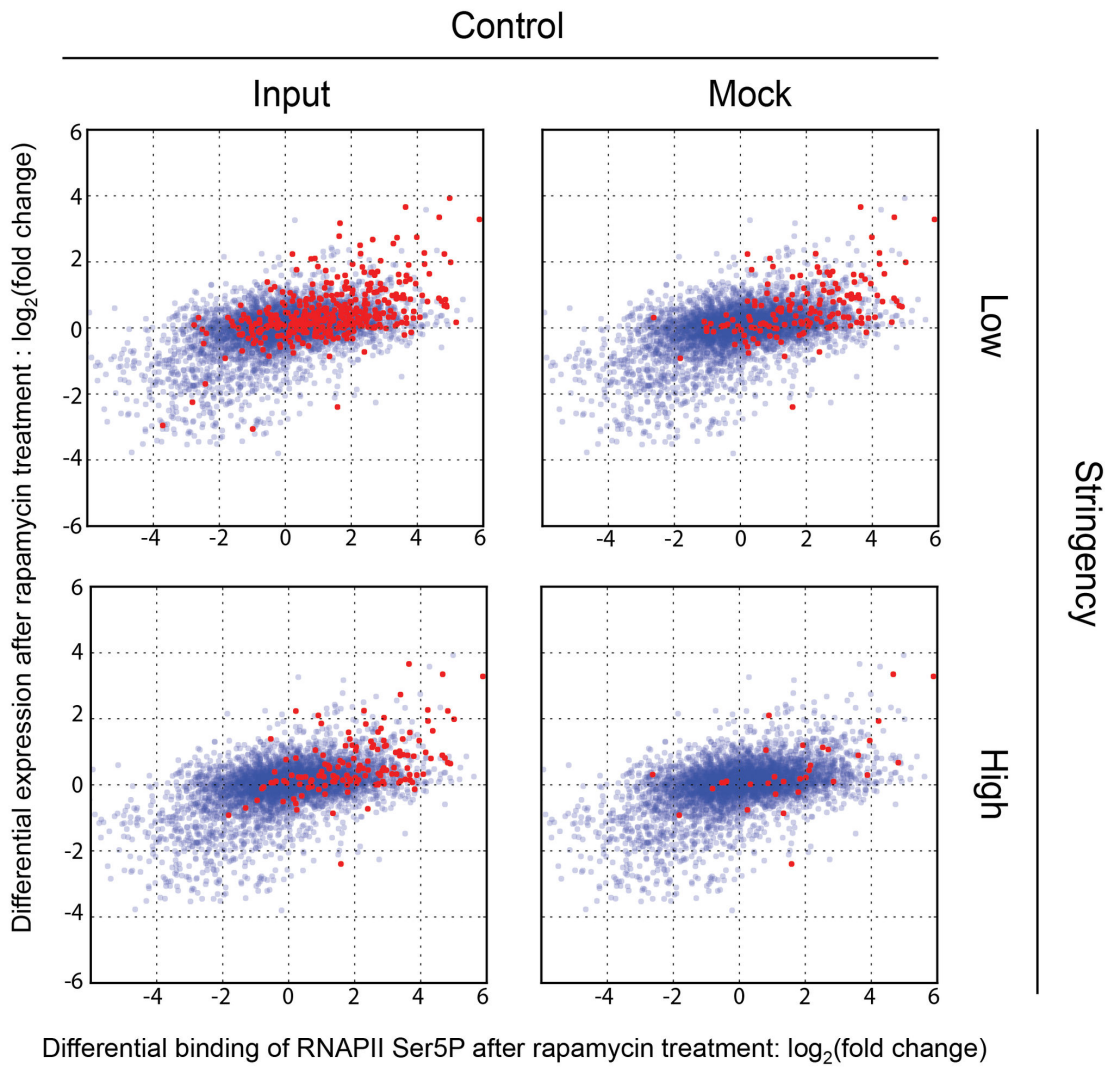
Mock ChIP is a better normalization control than input for minimizing false positive ChIP-seq targets. Either ChIP input or mock-ChIP was used as a control, at two q-value thresholds to obtain high and low significant peaks (see text and Materials and Methods). The scatter plots were drawn as described in Figure 4, and the numbers of Tup1 DBT ORFs (red) were as follows: input low stringency=379, input high stringency=139, mock low stringency=149, mock high stringency=32.

The use of mock ChIP as a normalization control resulted in a lower proportion of DBT ORFs (49.2% vs 36.6% and 36.2% vs 19.4% in Table 1), suggesting that mock ChIP is a more effective normalization control for expression bias than the input sample. The use of a more stringent threshold in conjunction with a mock ChIP normalization control reduced the number of DBT ORFs that were correlated with high transcription rates in an obvious manner (Figure 5). However, even this method of minimizing such likely false positives is not infallible. For example, *GAP1* and *ASN1* were activated by rapamycin and showed Tup1 occupancy signals that were comparable to true peaks (Figure S3). *GAP1* expression increased by 3.64 fold in a *tup1Δ* strain compared to WT, strongly suggesting that Tup1 is a repressor, rather than an activator of *GAP1*. Establishing a role for Tup1 in activating these genes in response to rapamycin is therefore non-trivial. Thus, while mock ChIP is a more stringent control for the identification of Tup1 DBTs in response to rapamycin, there is still strong evidence for apparent differential binding to several ORFs, where it is difficult to distinguish between expression bias or true binding with biological significance.

### Careful interpretation is required when drawing conclusions about transcription rates from the strength of ChIP peak signals

**Figure 6:**
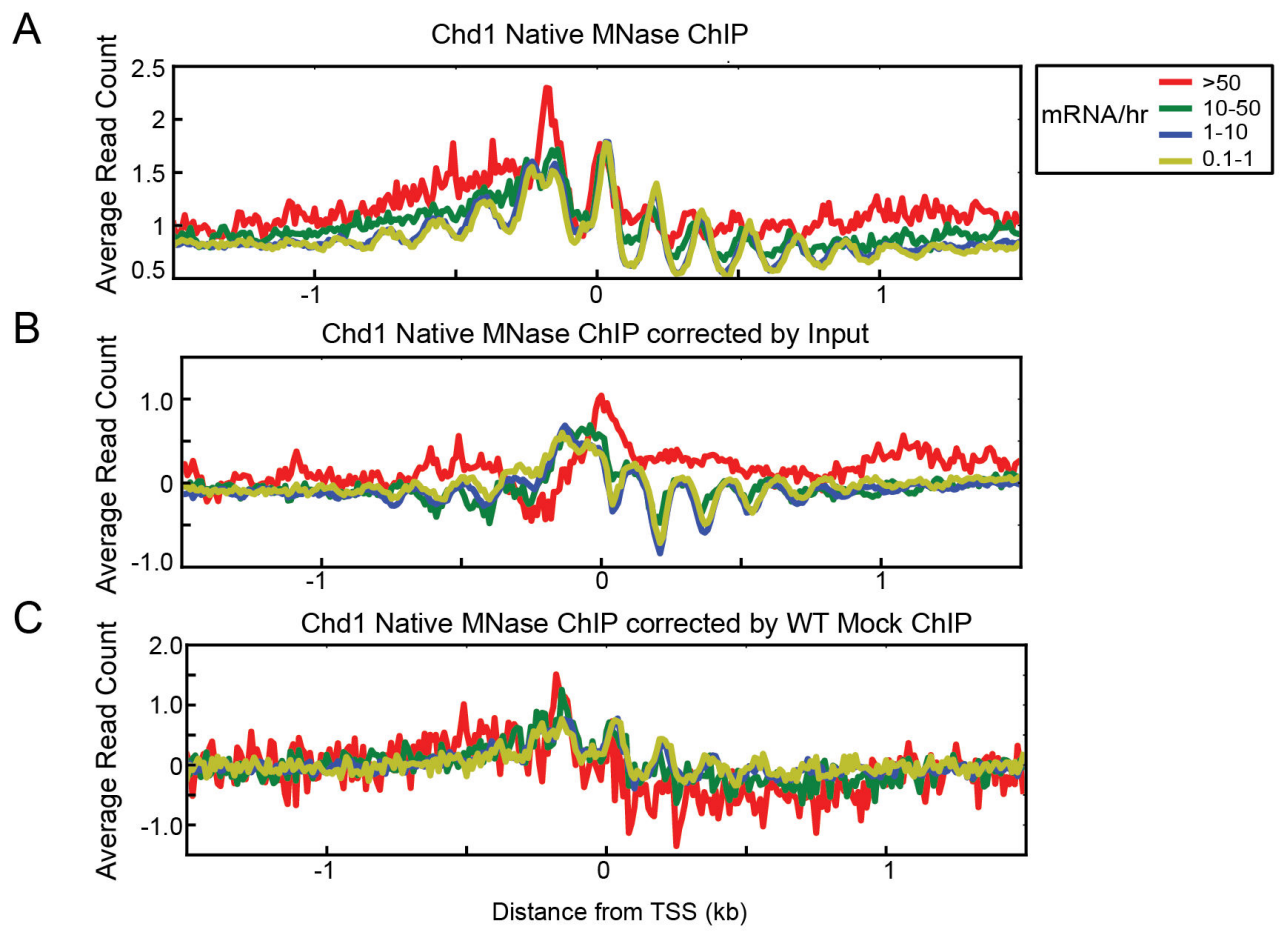
Expression bias may provide a misleading picture of the relationship between ORF binding and transcription rate. (A, B) Previously published ChIP-seq data for Chd1 [33] was plotted either uncorrected (A) or corrected by input (B). Occupancy is higher at high TR genes compared to low TR genes, when genes are ranked by mRNA/hr [45]. (C) Same Chd1 ChIP-seq data, after correction by mock ChIP-seq data, no longer shows a strong relationship of Chd1 occupancy with transcription rate.

Unlike sequence-specific transcription factors, ChIP for chromatin remodelers and chromatin-modifying enzymes is inherently difficult because of how transiently these factors bind to chromatin [14]. Many chromatin remodeler ChIPs demonstrate weakly detectable signals to begin with, making it harder to distinguish them from expression bias. To investigate the effect of expression bias in chromatin remodeler ChIPs, we examined MNase ChIP-seq data for the ATP-dependent remodeler Chd1 from a previously published paper reporting that Chd1 associated with the transcribed regions of actively transcribed genes [33]. We mapped these reads with BWA and discarded non-uniquely mapped reads because the paired-end reads had a read length of only 25 bases. We plotted the read profile relative to yeast transcription start sites and observed the nucleosomal periodicity expected for the association of chromatin remodelers with chromatin. As reported, Chd1 occupancy on high TR genes was higher than other gene groups both before and after input correction (Figure 6A and B). However, the difference in Chd1 occupancy between high TR genes and low TR genes was very small using input correction. When we normalized Chd1 occupancy with our mock ChIP data, however, the correlation with the transcription rate was no longer observed (Figure 6C). Thus, the association of Chd1 binding to ORFs and its relationship with transcription rate remains unclear when expression bias is properly accounted for.

### Expression bias suggests directionality of transcription activated from divergently regulated promoters

**Figure 7:**
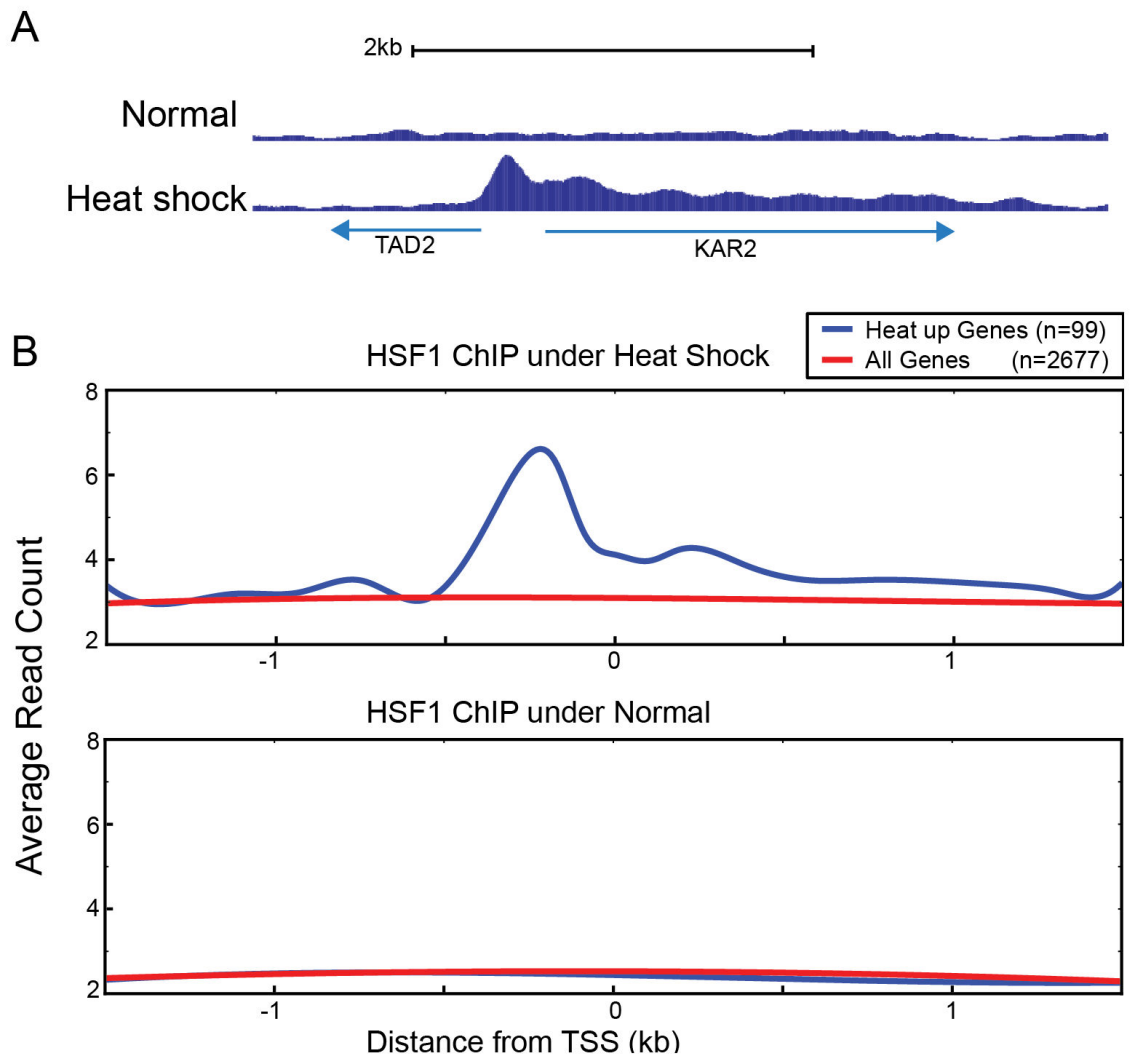
ChIP-seq signal from binding of Hsf1 to bidirectional promoters is asymmetrically skewed towards Heat Up genes. (A) Hsf1 strongly bound to the shared promoter of TAD2 and KAR2. The binding signals gradually decreased towards the 3’ end of KAR2 which is strongly activated upon heat shock, whereas the signal dropped sharply in the direction of TAD2 transcription. (B) Average Hsf1 occupancy over the 99 divergent genes out of the top 200 Heat Up genes (red), and all the other divergent genes (blue) under normal and heat shock conditions. In this representation, Heat Up genes were arranged on the right with respect to the genes whose promoter was shared, which reveals that Hsf1 binding decreases gradually over the Heat Up genes.

When a transcriptional activator binds to bidirectional (divergently regulated) promoters, it can be difficult to identify which of the two divergent ORFs, if any, is transcriptionally regulated by its binding. We examined ChIP-seq data for Hsf1 to see if expression bias could shed light on this issue. HSF1 is a key activator of the transcriptional response to heat shock, strongly binding to the promoters of the Heat Up genes after heat shock [34]. We noticed that the signal for Hsf1 binding was asymmetric across the two divergent ORFs. The peak of Hsf1 binding occurred between the start sites of *TAD2* and *KAR2* but the tail of the Hsf1 ChIP-seq signal extended toward *KAR2*, not *TAD2* (Figure 7A). Based upon the differential binding of RNAPII Ser5P after heat shock, *KAR2* was strongly transcriptionally activated, while *TAD2* was not. 99 genes out of the top 200 RNAPII Ser5P heat shock DBTs shared promoters with another divergently transcribed gene. At these genes, the tails of Hsf1 binding stretched toward the DBTs (Figure 7B). Thus, the ChIP-seq binding signals over ORFs for transcription factors that strongly activate gene expression can potentially identify the correct target gene from bidirectionally transcribed ORFs.

### The expression bias is amplified during library construction

**Figure 8:**
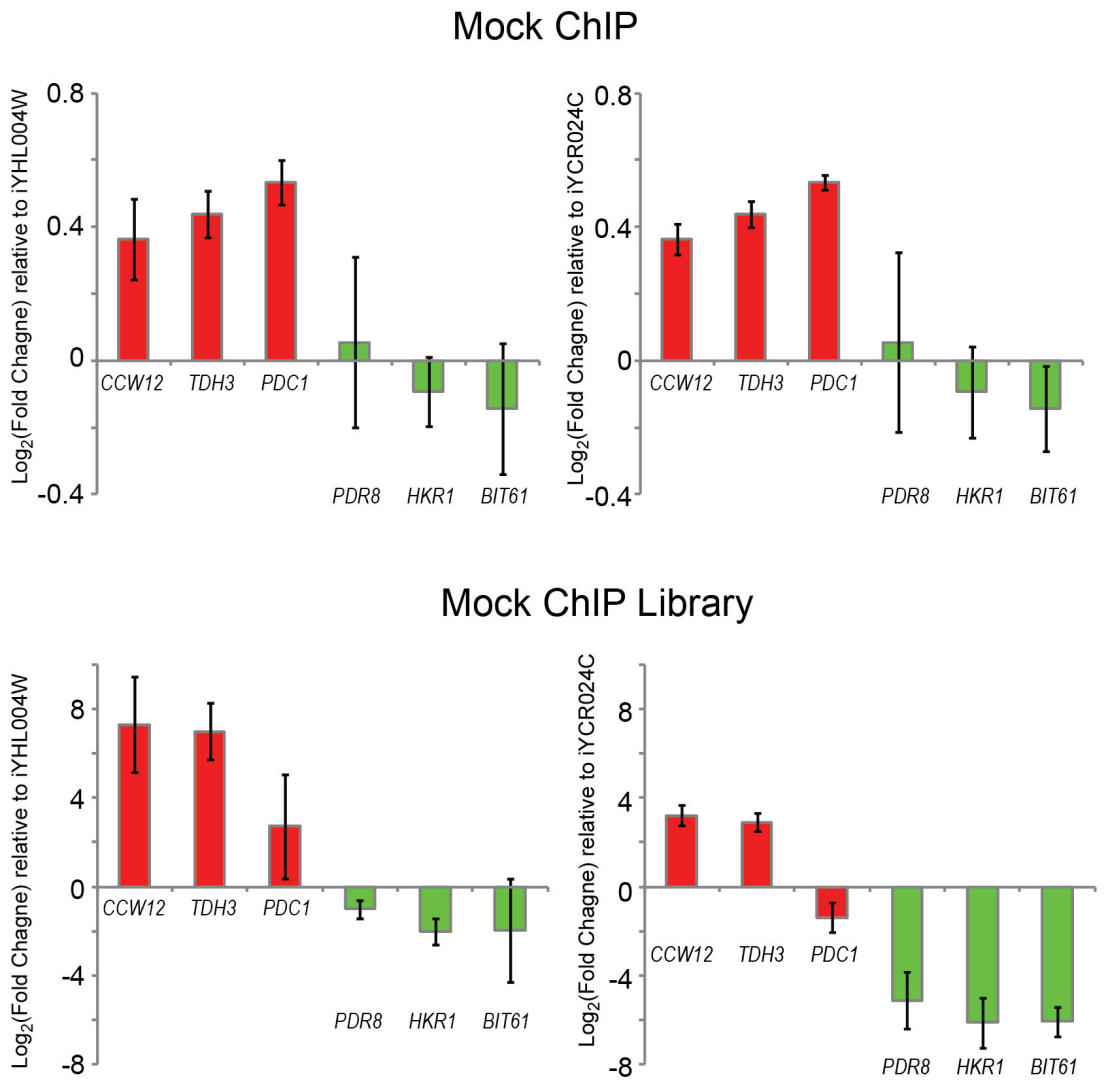
qPCR shows higher expression bias in sequencing library than mock ChIP. Three ORFs showing high enrichment of RNAPII Ser5P and high expression levels by RNA-seq were selected as high TR genes, shown in red (CCW12, TDH3, and PDC1). Three ORFs were picked as low TR genes using the same criteria, and are shown in green (PDR8, HKR1, and BIT1). For relative quantification of targets, two different controls were used (iYHL004W, plotted on left and iYCR024C, plotted on right), and fold-changes were calculated by dividing the mean of target quantities by the mean of control quantities. Three biological replicates were carried out with two independently prepared mock ChIP samples, one of which was used for sequencing. Error bars represent the standard deviation of three log_2_-transformed fold change values from the replicate experiments. Data from individual replicate experiments are shown in Figure S6.

To establish whether the expression bias is primarily an artifact arising during sequencing library construction procedures or already exists in the immunoprecipitated DNA, we carried out quantitative PCR using ChIP-ed DNA before and after library construction. As examples of genes showing the expression bias, we chose three genes, *CCW12*, *TDH3*, and *PDC1*, which had the highest expression bias based on the mock ChIP read counts and also ranked within the top 20 most highly expressed genes based on read counts from RNA-seq and RNAPII Ser5P ChIP-seq. As negative targets, we selected *PDR8*, *HKR1*, and *BIT6* as they had low read counts in mock ChIP, RNA-seq, and RNAPII Ser5P ChIP-seq. In mock ChIP DNA, the genes showing high expression bias were overrepresented, whereas the genes showing no expression bias were underrepresented, indicating that the expression bias was present even before sequencing libraries were made (Figure 8). In the sequencing libraries, these differences in representation were magnified (Figure 8), indicating that amplification during sequencing library construction could result in the over-representation of high TR genomic regions in sequencing results.

### Nucleosomal periodicity of RNAPII Ser5P ChIP is corrected by input

**Figure 9:**
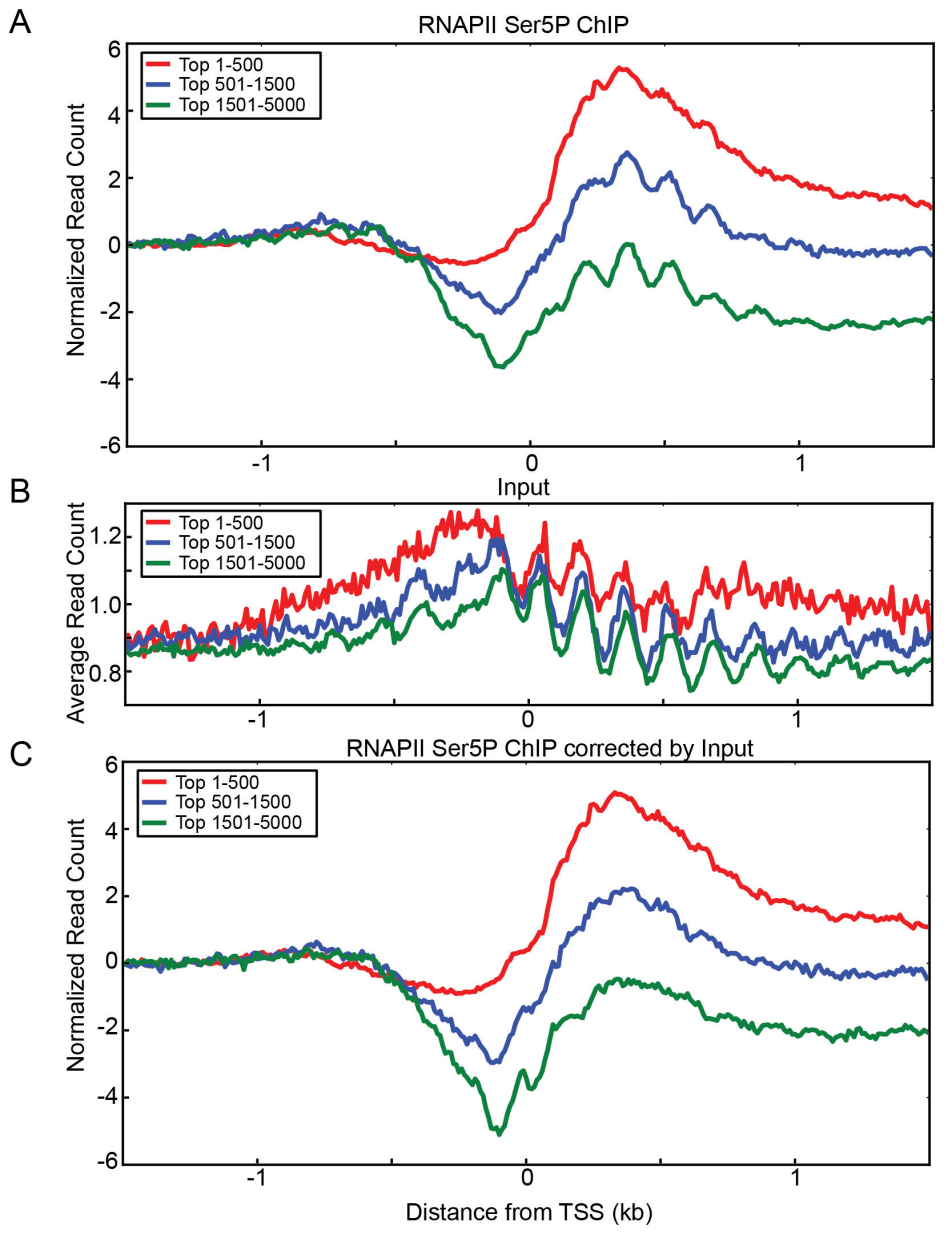
Nucleosomal periodicity in a ChIP-seq dataset can be corrected with input correction. ORFs were grouped by RNAPII Ser5P occupancy into 3 categories as indicated, and normalized sequencing reads of the 3 categories are shown on the Y-axis. (A) In the RNAPII Ser5P ChIP-seq read profiles, low expressed genes (blue and green lines) exhibited nucleosomal periodicity. Occupancy within each set was independently scaled and the profiles were set to start at the zero position on the Y-axis. (B) Input shows strong nucleosomal periodicity although average signal intensity is low (C) By subtracting input signal from RNAPII Ser5P signals, the apparent nucleosomal periodicity of RNAPII Ser5P was greatly reduced. Scaling factor for each group of genes was the same as in A.

We observed that many ChIP-seq profile plots showed a periodicity of mean read counts over regions devoid of strong peaks (Figure 2 and Figure S1). This periodicity within gene bodies, which was identical to nucleosomal periodicity, was also present in RNAPII Ser5P ChIP and especially noticeable for low TR genes (Figure 9A). The naïve interpretation of these data would be that active RNAPII binds to individual nucleosomes and/or that RNAPII stalls at the center of nucleosomes during transcription. However, this interpretation, solely based on this observation would be misleading because even input exhibited similar strong periodicity (Figure 9B), as did Tup1 and Swi6 (Figure 2 and S1). When we normalized the RNAPII Ser5P read counts by the input read counts for each corresponding gene, the nucleosomal periodicity of the RNAPII Ser5P ChIP-seq was eliminated (Figure 9C), indicating that this periodicity was not a true signal but rather another artifact.

## Discussion

In the analysis of ChIP-seq data, two types of normalization or correction controls are commonly used: mock ChIP and input DNA. The input sample has the advantage that all the regions of the genome are well represented, the sample concentration is ample and stable for constructing sequencing libraries, and the same sample can potentially serve as the control for several related experiments. The input generates a baseline signal for reads across the genome, factoring in sequence mappability and copy number differences relative to the reference genome. For these reasons, input has been suggested as a more effective control [35]. However, our results show that a background signal deriving from expression bias, namely genes transcribed at high rates, is not adequately represented in the input (Figure 2C). A mock ChIP sample processed in parallel through the immunoprecipitation and subsequent steps better reflects the background enrichment from highly transcribed genes and therefore is a better control for minimizing the appearance of occupancy signal over transcribed regions. However the DNA yield after a mock ChIP step is typically lower and likely to be more variable from experiment to experiment.

It is often assumed that measuring the binding of a transcription factor under two different conditions and identifying the differentially bound targets (DBTs) offers the most reliable way to identify targets of biological significance. This assumes that most sources of background signal are canceled out between the two samples in such an experimental strategy. Our results indicate that this assumption is risky. Because the expression bias derives directly from actively transcribed genes, and transcription will differ between the two conditions, it will appear as if the factor under study shows differential binding when in fact it is the background expression bias that is differently represented in the two conditions. We suggest therefore that even in these cases, the ChIP data from each condition has to be properly corrected by the corresponding mock ChIP data to minimize false positives.

The expression bias we demonstrate has the potential to skew ChIP-seq data into representing any chromatin-associated protein as being associated with gene bodies or ORFs in yeast, regardless of the protein’s true role. In particular, this misinterpretation is easy to arrive at when the proteins of interest are ones that often show low signal strength in ChIP-seq experiments, such as chromatin remodelers, histone modifying or associated factors, or components of the general transcription machinery [33,36,37].

It is beyond the scope of this study to definitively identify the source and mechanism of this background expression bias in ChIP-seq data. However, given that it is most strongly observed at highly transcribed genes, we speculate that in many cases it arises from direct or indirect non-specific interactions of the immunoprecipitated protein with DNA in open chromatin at highly transcribed regions, trapped by the crosslinking process. It is unclear why the phenomenon exists even in mock ChIP datasets, where there is no expected interaction between the non-specific antibody and any cellular protein that might interact with DNA. Here, it is possible that even low level non-specific interactions between the antibody and cross-reacting cellular proteins contribute to this phenomenon, or that open chromatin shows preferential recovery through the immunoprecipitation process. Indeed, the latter property underlies methods such as FAIRE and Sono-seq, which are aimed at globally recovering open chromatin regions [38,39].

This pattern of the Hsf1 ChIP-seq signal is informative with regard to how background peaks derived from expression bias might be related to true occupancy in some cases. The strong background starts just downstream of the true Hsf1 binding site and gradually tapers off toward the 3’ end of the gene (Figure 7A and B, Figure S4). This tail structure suggests a model in which high TR genes that are opened by the transcription process facilitate the expression bias. The transcription machinery and co-factors are recruited onto the open chromatin of heat-activated genes upon heat shock in conjunction with Hsf1 recruitment. The close proximity of Hsf1 to this transcription machinery can allow them to be crosslinked and co-immunoprecipitated. We speculate that this proximity effect of Hsf1 around open chromatin generates the tail structure observed. The expression bias in the other ChIP data may similarly be derived from these open chromatin interactions. Importantly, to the extent that the expression bias is always related to transcriptional activity, and will be observed most strongly when a transcription factor capable of interacting with chromatin is immunoprecipitated after crosslinking, this background is essentially indistinguishable from true "biological" targets, especially when the true targets are seen at low levels. Our data address this phenomenon only in yeast ChIP-seq data, but conceivably, this could extend to ChIP-seq experiments in other eukaryotes as well. For example in mammals, cell-type or tissue-specific open chromatin is known to occur at promoters and enhancers [40]. A similar phenomenon as we described here for yeast could in part explain observations of hotspots of transcription factor binding and instances of neutral transcription factor binding, where such apparent binding has no biological meaning [41,42].

The nucleosomal periodicity observed in input and non-target regions from ChIP may be the result of the high susceptibility of linker DNA to shearing. Linker DNA is not protected by histones and may be easier to break by shearing [43]. As a result, the ends of sheared DNA even in the input are more likely to be in linker DNA and have a higher chance of being ligated by sequencing adapters. The resulting sequenced fragments would show the nucleosomal periodicity that is typically observed in MNase-seq experiments (Figure S5). These low-level nucleosomal periodicity signals are not typically of concern in transcription factor ChIP because these experiments usually focus on stronger peaks at regulatory elements. However, the background nucleosomal periodicity may give a misleading picture when analyzing ChIP-seq against proteins that are localized within gene bodies, such as RNAPII-associated factors or chromatin remodelers, which do in fact associate with nucleosomes and/or demonstrate peaks in a similarly low range to the nucleosomal background. Our findings urge careful choice of ChIP-seq normalization controls and call for caution in interpreting the signals from ChIP-seq datasets showing transcription dependent occupancy of proteins over coding regions.

## Supporting Information

Figure S1 **High background signals at high TR genes in SOLiD sequencing data.** SWI6 Myc indicates ChIP against 13XMyc tagged Swi6 using c-Myc antibody conjugated agarose beads. We pulled down TAP tagged proteins for other ChIPs. The expression bias in TUP1 was the highest in SOLiD, and mock ChIP showed expression bias comparable to Swi6 ChIP.
(PDF)

Figure S2 **Two independent, previously published datasets exhibit similar expression bias.** We downloaded two previously published ChIP-seq datasets and ran our pipeline. 13XMyc tagged Cse4 was immunoprecipitated with the same beads as used in 13XMyc Swi6 ChIP in Figure S1 [13]. As a negative control ChIP for 13XMyc Tbf1 ChIP, monoclonal anti-Myc antibody was incubated with untagged W303-1A strain [23]. Both ChIP-ed DNA samples were sequenced using the Illumina platform.
(PDF)

Figure S3 **Examples of high expression bias in rapamycin-specific targets that are indistinguishable from true targets.** Based on differential binding of RNAPII Ser5P (DB) and differential expression by microarray (DE) in response to rapamycin, *GAP1* showed 59 and 10 positive fold-change in terms of DB and DE, respectively, ranking within the top 10 in both measurements. Although the rank of *ASN1* in DE was 1466, the DB was ranked in top 56 as 15 fold change. The gene bodies had strong signals for rapamycin-specific occupancy by Tup1, which could not be corrected by rapamycin-treated mock ChIP.
(PDF)

Figure S4 **Hsf1 occupancy signals stretches to the 3' end of up-regulated genes upon heat shock.** Average read counts of Hsf1 ChIP-seq were plotted for the top 200 up-regulated genes and all other genes, separately, without consideration of bidirectional/divergently transcribed promoters.
(PDF)

Figure S5 **Transcription depletes nucleosomes.** Both H3 MNase ChIP [24] and MNase-seq from our lab showed lower nucleosome occupancy in the top 100 highly transcribed genes under normal growth conditions.
(PDF)

Figure S6 **Relative quantification of genomic regions of high- and low-transcription in mock ChIP and the sequencing library by qPCR.** Three biological replicates of qPCR in Figure 8 are shown here individually. Bars with solid color, bars with slanted lines, and bars with dots were replicate 1, 2, and 3, respectively. Error bars represented the standard deviations of fold change derived from three technical replicates of each entity (target and control).
(PDF)

Table S1 **qPCR primers.**
(PDF)
