# Supplementary figures and images for: Widespread Misinterpretable ChIP-seq Bias in Yeast
Source: PLoS One. 2013 Dec 9;8(12):e83506. doi: 10.1371/journal.pone.0083506 (PMC3857294; doi:10.1371/journal.pone.0083506)

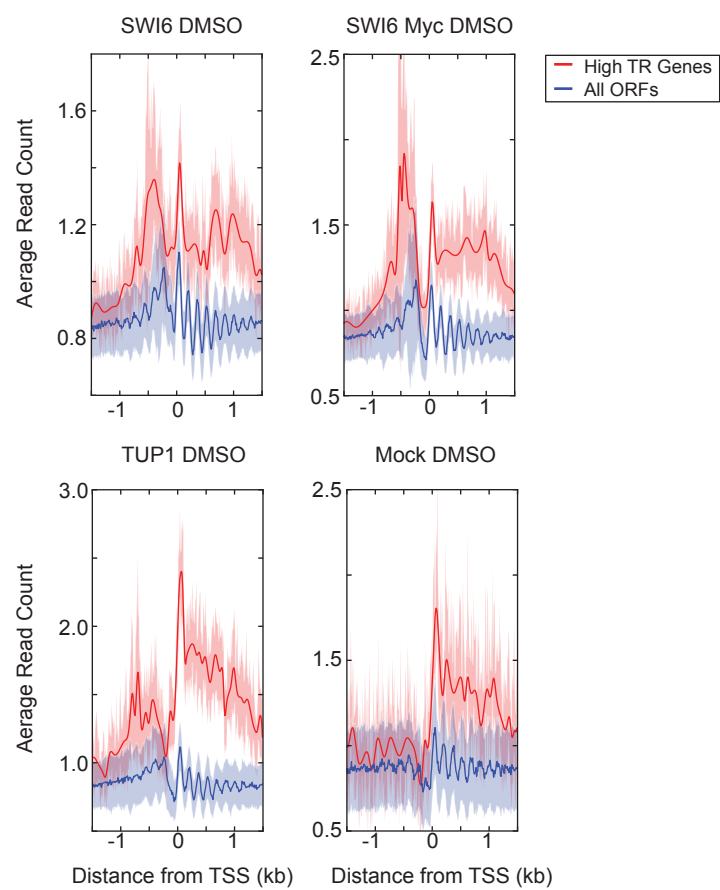

Supplement: Figure S1 — High background signals at high TR genes in SOLiD sequencing data. SWI6 Myc indicates ChIP against 13XMyc tagged Swi6 using c-Myc antibody conjugated agarose beads. We pulled down TAP tagged proteins for other ChIPs. The expression bias in TUP1 was the highest in SOLiD, and mock ChIP showed expression bias comparable to Swi6 ChIP. (PDF) [file pone.0083506.s001.pdf]

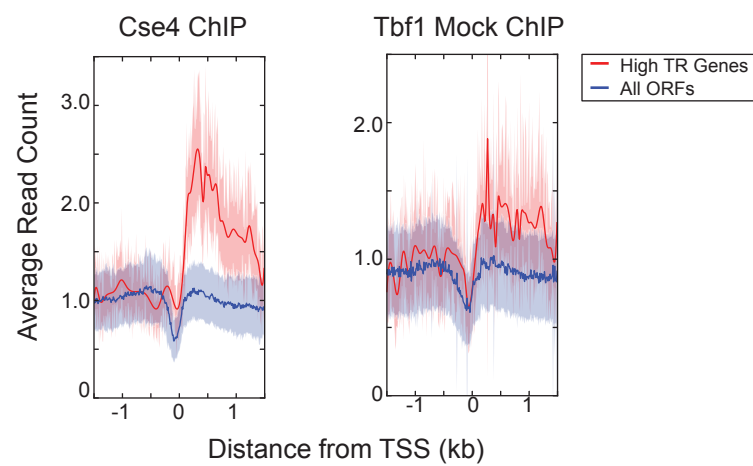

Supplement: Figure S2 — Two independent, previously published datasets exhibit similar expression bias. We downloaded two previously published ChIP-seq datasets and ran our pipeline. 13XMyc tagged Cse4 was immunoprecipitated with the same beads as used in 13XMyc Swi6 ChIP in Figure S1 [13]. As a negative control ChIP for 13XMyc Tbf1 ChIP, monoclonal anti-Myc antibody was incubated with untagged W303-1A strain [23]. Both ChIP-ed DNA samples were sequenced using the Illumina platform. (PDF) [file pone.0083506.s002.pdf]

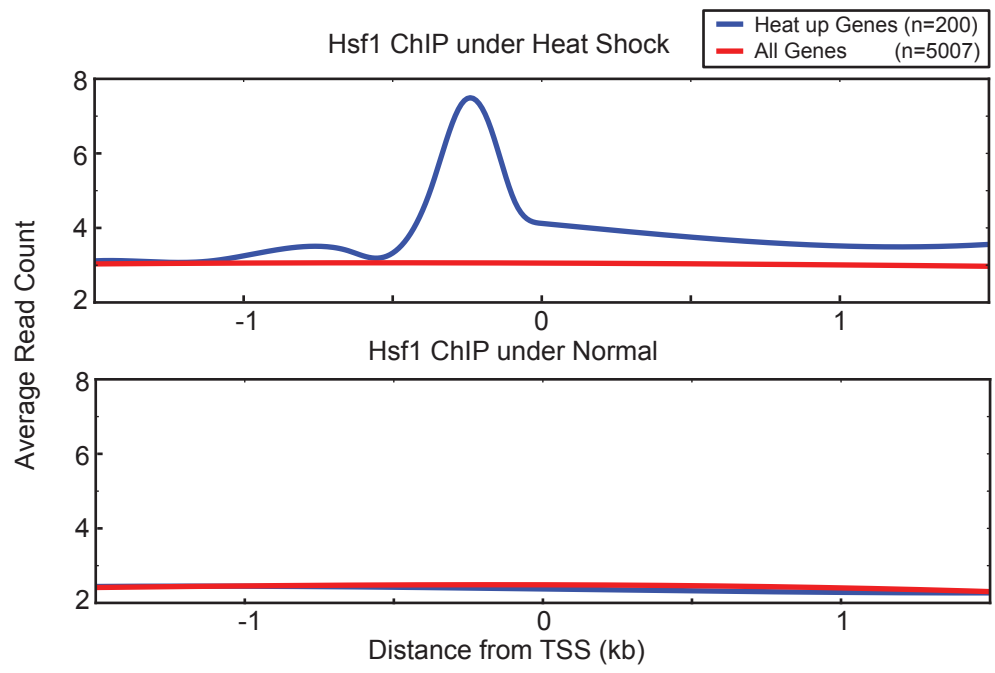

Supplement: Figure S4 — Hsf1 occupancy signals stretches to the 3' end of up-regulated genes upon heat shock. Average read counts of Hsf1 ChIP-seq were plotted for the top 200 up-regulated genes and all other genes, separately, without consideration of bidirectional/divergently transcribed promoters. (PDF) [file pone.0083506.s004.pdf]

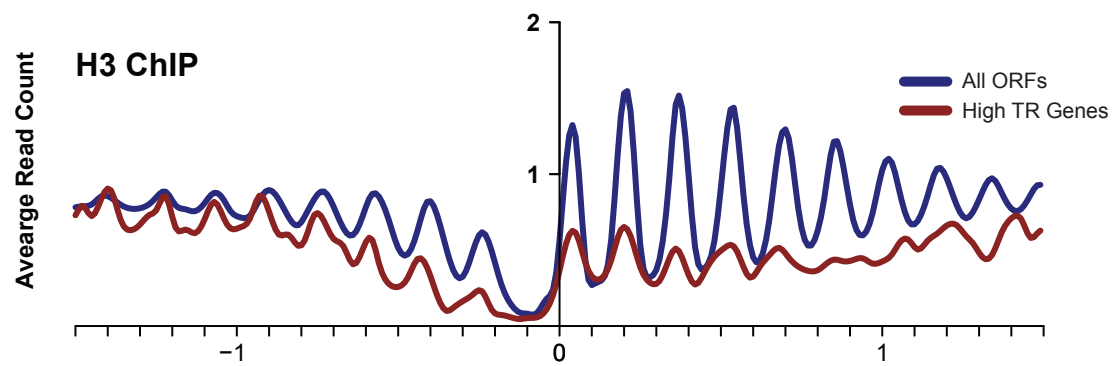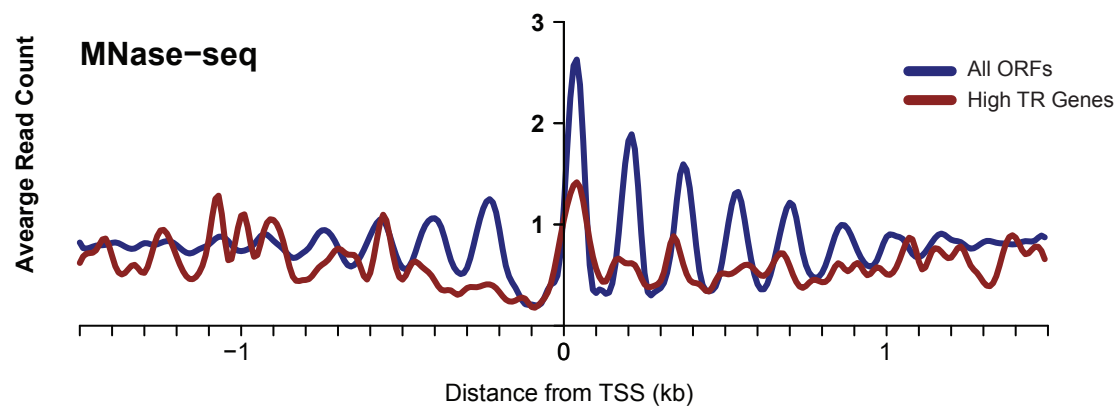

Supplement: Figure S5 — Transcription depletes nucleosomes. Both H3 MNase ChIP [24] and MNase-seq from our lab showed lower nucleosome occupancy in the top 100 highly transcribed genes under normal growth conditions. (PDF) [file pone.0083506.s005.pdf]

## Mock ChIP

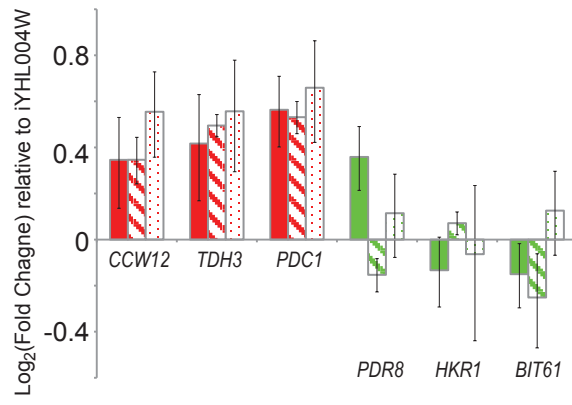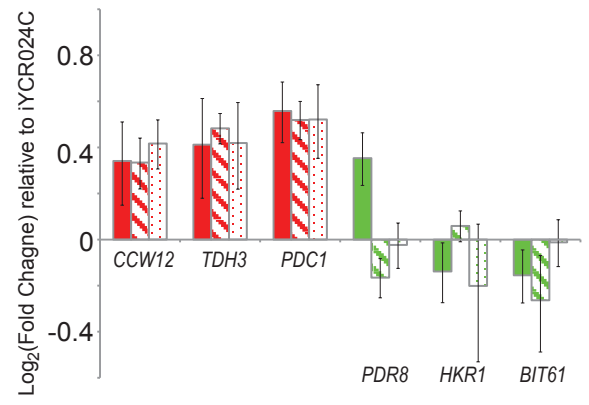

## Mock ChIP Library

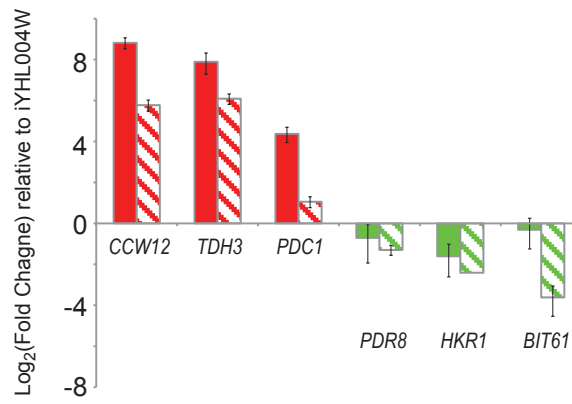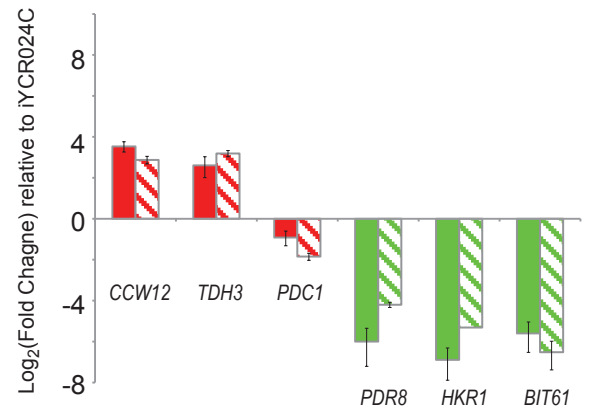

Supplement: Figure S6 — Relative quantification of genomic regions of high- and low-transcription in mock ChIP and the sequencing library by qPCR. Three biological replicates of qPCR in Figure 8 are shown here individually. Bars with solid color, bars with slanted lines, and bars with dots were replicate 1, 2, and 3, respectively. Error bars represented the standard deviations of fold change derived from three technical replicates of each entity (target and control). (PDF) [file pone.0083506.s006.pdf]
