# Supplementary material for: Widespread Misinterpretable ChIP-seq Bias in Yeast
Source: PLoS One. 2013 Dec 9;8(12):e83506. doi: 10.1371/journal.pone.0083506 (PMC3857294; doi:10.1371/journal.pone.0083506)
Supplement: Table S1 — qPCR primers. (PDF) [file pone.0083506.s007.pdf]

Table S1. qPCR primers

name	Forward sequence	Reverse sequence
CCW12	CATTGACCACTGAAGCCCCA	ACAGAGTGAGTTGGAGCAGC
TDH3	GTTTCCCACGATGACAAGCAC	TCAACGTTGGAAGAACCCCA
PDC1	ACACCATCTTGGCTTTGGTCA	CCTTGACGTCGTGTCTGGAA
PDR8	GAGAGCCCGTTTCAAGTGGT	TTGCTGACACATAGGCCTGG
HKR1	ATTGGACCTGCTACGACTGC	TTTGGAGAGGGGTACGGAGT
BIT61	GGCAACTTGTGGACTCAGGA	CGGTGAGGCAGGATCAGTTC
iYHL004W	GCCAACCCGAAGACGCTAAA	AGAAGCATAAACCCAAGTACGAA
iYCR024C	AACATAGGTCAGGCCGAATG	AGCATTTTTCTTCAATATGAAGGTACT
