# Supplementary material for: Widespread Misinterpretable ChIP-seq Bias in Yeast
Source: PLoS One. 2013 Dec 9;8(12):e83506. doi: 10.1371/journal.pone.0083506 (PMC3857294; doi:10.1371/journal.pone.0083506)
Supplement: Figure S3 — Examples of high expression bias in rapamycin-specific targets that are indistinguishable from true targets. Based on differential binding of RNAPII Ser5P (DB) and differential expression by microarray (DE) in response to rapamycin, GAP1 showed 59 and 10 positive fold-change in terms of DB and DE, respectively, ranking within the top 10 in both measurements. Although the rank of ASN1 in DE was 1466, the DB was ranked in top 56 as 15 fold change. The gene bodies had strong signals for rapamycin-specific occupancy by Tup1, which could not be corrected by rapamycin-treated mock ChIP. (PDF) [file pone.0083506.s003.pdf]

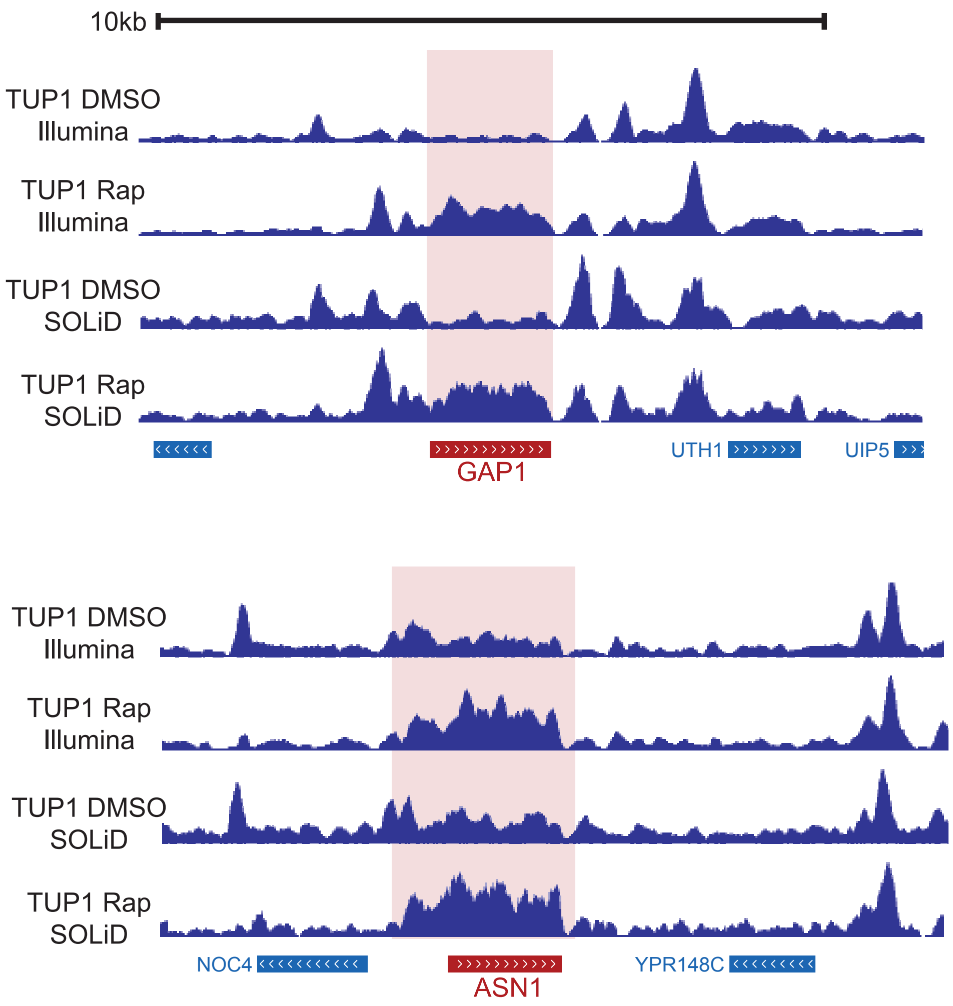
10kb
TUP1 DMSO Illumina
TUP1 Rap Illumina
TUP1 DMSO SOLiD
TUP1 Rap SOLiD
GAP1
UTH1
UIP5
TUP1 DMSO Illumina
TUP1 Rap Illumina
TUP1 DMSO SOLiD
TUP1 Rap SOLiD
NOC4
ASN1
YPR148C
